# Early detection of liver injuries by the Serum enhanced binding test sensitive to albumin post-transcriptional modifications

**DOI:** 10.1038/s41598-024-51412-0

**Published:** 2024-01-16

**Authors:** Souleiman El Balkhi, Mohamad Ali Rahali, Roy Lakis, François Ludovic Sauvage, Marving Martin, Angelika Janaszkiewicz, Roland Lawson, Ruben Goncalves, Paul Carrier, Veronique Loustaud-Ratti, Anne Guyot, Pierre Marquet, Florent Di Meo, Franck Saint-Marcoux

**Affiliations:** 1 P&T, UMR1248, Inserm, Univ. Limoges, Limoges, France; 2 Department of Pharmacology, Toxicology and Pharmacovigilance, CHU Limoges, Limoges, France; 3 Department of Liver Disease, CHU Limoges, Limoges, France; 4 Department of Pathology, CHU Limoges, Limoges, France; 5 Pharmacology-Toxicology and Pharmacovigilance Department, Centre de Biologie Et de Recherche en Santé (CBRS), 2, Av. Martin Luther King, 87042 Limoges Cedex, France

**Keywords:** Metals, Proteins, Proteomics

## Abstract

Early and sensitive biomarkers of liver dysfunction and drug-induced liver injury (DILI) are still needed, both for patient care and drug development. We developed the Serum Enhanced Binding (SEB) test to reveal post-transcriptional modifications (PTMs) of human serum albumin resulting from hepatocyte dysfunctions and further evaluated its performance in an animal model. The SEB test consists in spiking serum ex-vivo with ligands having specific binding sites related to the most relevant albumin PTMs and measuring their unbound fraction. To explore the hypothesis that albumin PTMs occur early during liver injury and can also be detected by the SEB test, we induced hepatotoxicity in male albino Wistar rats by administering high daily doses of ethanol and CCl_4_ over several days. Blood was collected for characterization and quantification of albumin isoforms by high-resolution mass spectrometry, for classical biochemical analyses as well as to apply the SEB test. In the exposed rats, the appearance of albumin isoforms paralleled the positivity of the SEB test ligands and histological injuries. These were observed as early as D3 in the Ethanol and CCl_4_ groups, whereas the classical liver tests (ALT, AST, PAL) significantly increased only at D7. The behavior of several ligands was supported by structural and molecular simulation analysis. The SEB test and albumin isoforms revealed hepatocyte damage early, before the current biochemical biomarkers. The SEB test should be easier to implement in the clinics than albumin isoform profiling.

## Introduction

Post-translational modifications (PTMs) of Human serum albumin (HSA) have gained interest recently as biomarkers of advanced liver diseases^1–11^. HSA may undergo several PTMs including truncations, acetylation, cysteinylation, homocysteinylation, glutathionylation, glycation, nitrosylation, nitration, phosphorylation and oxidation^12^. They translate in modifications of albumin binding properties^5^. This aspect has been exploited by Bar-Or et al., who proposed the albumin cobalt binding test (ACB) also known as the Ischemia Modified Albumin test (IMA) to detect cardiac ischemia^6^. The IMA test is based on the fact that cardiac ischemia is associated with modifications in the structure of albumin and, thus, in the capacity of a specific binding site to bind cobalt. Since the approval of the IMA as a biomarker of cardiac ischemia by the FDA (Regulation number: 862.1215; http://www.accessdata.fda.gov), this test has also been investigated in liver diseases showing correlation with the severity of cirrhosis^7,13–15^. Briefly, the IMA test is performed by adding CoCl_2_ and dithiothreitol to serum, followed by colorimetric measurement of the (free-Co)-dithiothreitol complex at 470 nm.

Thereafter, the clinical relevance of other albumin modifications has been investigated in advanced liver diseases ^10,12^. PTMs related to Cys34 oxidation have been the most studied. They are characterized on the basis of the redox state of Cys34 as follows: (i) Human mercaptalbumin (HMA), also known as reduced HAS or native HSA (70 -80% of total HAS in healthy subjects); (ii) Nonmercaptalbumin 1 (HNA1), a reversibly oxidized form (20–30%); and (iii) Nonmercaptalbumin 2 (HNA2), an irreversibly oxidized form of albumin (< 5%) ^12^. The increase in HNA1 and HNA2 has been documented in end-stage liver pathologies, with a progressive increase of these isoforms in severe cirrhosis that is associated with a very high short-term mortality^4,9,10^. PTMs involving sites others than Cys34 were also reported. N- or C-terminal truncated, as well as glycated, forms were found in plasma samples from patients with acutely decompensated cirrhosis or severe alcoholic hepatitis^4^. Interestingly, HNA1 played a pejorative role in decompensated cirrhosis^16^, whereas native HSA had a protective role by reducing the proinflammatory environment present in patients with acutely decompensated cirrhosis^17^. At present, it is not fully established whether other albumin isoforms possess yet uncharacterized biological properties.

This bundle of arguments strongly suggests that albumin modifications detected in blood may reflect the dys/function of hepatocytes and could represent a versatile tool for the diagnosis and the prognosis of liver injuries and/or diseases. Since HSA is continuously and exclusively synthesized and matured in the liver, albumin modifications may be directly related to the chemical environment into the hepatocytes, hence any liver dysfunction. Also, due to its peculiar structure, its abundance in blood (60% of all proteins), to the multiplicity of its ligands and binding sites and its role as the primary scavenger, it is now clear that not only the quantity, but also the quality of albumin could impact its physiological roles.

We here hypothesize that PTMs occur at early stages of liver injuries. Furthermore, given that some PTMs locally modify albumin structure, we assume that modulations of binding properties of HSA can be associated with different structural dynamics. This can in turn be indirectly revealed by investigating HSA binding capacity for different ligands. Interestingly, it was reported that each of the following ligands has a specific binding site on HSA: (i) gold (Au) binds preferentially to Cys34; (ii) copper (Cu) to the N-terminal binding site; (iii) cadmium (Cd) to the multi-metal binding site; (iv) L-thyroxine up to 5 specific binding sites and (v) dansylsarcosine to drug site 2^8^.

On these premises, this study aimed to: (i) develop the serum enhanced binding (SEB) test, using all the ligands mentioned above, as a functional test of PTMs and an early biomarker of liver dysfunctions; (ii) evaluate the SEB test as well as albumin PTMs in an animal model repeatedly exposed to toxic doses of ethanol and Carbon tetrachloride (CCl_4_); (iii) investigate the impact of PTMs onto the structure and dynamics of albumin, (iv) interpret the results of the SEB test in light of albumin PTMs, classic hepatic laboratory tests and liver histology.

## Materials and methods

### Chemicals

The ligand solutions were prepared using reagents purchased from Sigma-Aldrich: cobalt(II) chloride (CAS: 7646-79-9), gold(III) chloride trihydrate (CAS: 16961-25-4), copper(II) chloride (CAS: 7447-39-4), silver acetate (CAS: 563-63-3), and L-thyroxine sodium salt pentahydrate (CAS: 6106-07-6). Dansylsarcosine Piperidinium Salt (> 95%) was obtained from RareChemicals GmbH. All ligands were diluted with MilliQ purified water, and their pH levels were measured before use. Human albumin Vialebex, 200 mg/mL, was utilized to test binding capacity.

### Setting up the Serum Enhanced Binding (SEB) test

#### Patients

We collected residual serum samples from 90 patients admitted to Limoges University Hospital, following their consent for the use of residual biological materials, in accordance with local and French regulations (Code de la Santé Public, Art. L1211-2) and the declaration of Helsinki for experiments involving human subjects. The study was coordinated by the University Hospital of Limoges and the biocollection was authorized by the French Ministry of Health and registered under numbers DC 2010-1074 and AC-2016-2758, in accordance with the French Bioethics Act 2011-814 of July 7, 2011. Informed consent was obtained from all the patients.

Among the 90 samples collected, 45 were obtained from control patients without hepatic impairment, and the remaining 45 were from patients at various stages of cirrhosis It's important to note that the control patients were patients from various department form our hospital distinct from the liver disease unit and that they had other underlying medical conditions. The serum samples, collected in dry tubes and sent to the laboratory for routine biochemical tests, were used in this study after the completion of routine analyses. Control patients were included when their clinical diagnosis did not indicate any liver dysfunction, and their levels of transaminases (AST, ALT), alkaline phosphatases (ALP), gamma-glutamyl transferases (GGT), total and conjugated bilirubin (BILIT, BILID), and lactate dehydrogenase (LDH) were within normal ranges. Cirrhotic patients, classified as cirrhosis A, B, or C using Child–Pugh scores, were diagnosed according to BAVENO VII recommendations^18^.

#### Analytical procedures

*Ligands optimization*: We first evaluated the global capacity of serum to bind Cu, Au, L-thyroxine, Cd and dansylsarcosine in patients with no liver dysfunction. Each ligand was independently added in increasing concentrations to patient serum samples in order to obtain HSA/ligand theoretical ratios (mol/mol) of 1/1, 1/5, 1/10, 1/20, 1/50, 1/100, 1/500, and 1/1000 when possible. These theoretical ratios were calculated with 0.6 mM as an average concentration HSA in the serum.

Six different serum samples (from six different patients) per ligand and per ratio were used for this evaluation. After incubation for 30 min at 4 °C, the serum samples were ultrafiltrated on Amicon® filters with a 30 kDa cut-off and 10 µL of the ultrafiltrate was then diluted in HNO_3_ 0.1 M before analysis using a multi-element ICP-MS method for the determination of free (unbound) concentrations of Cu, Au, Cd, iodine (for L-Thyroxine) and sulfur (for dansylsarcosine). The bound fractions as well as the concentration ratios of HSA/bound ligand (mol/mol) were then calculated.

To confirm that the binding is only due to HSA and that there is no unspecific binding, we performed the same tests on a commercial human albumin solution Vialebex®, 200mg/mL. This allowed us to determine for each ligand its maximum unspecific binding capacity. These thresholds were then set to best discriminate between serum samples containing mostly modified HSA or mostly native HSA.

*Comparison of HSA binding capacities in patients with liver cirrhosis or no hepatic dysfunction*: After establishing the threshold for > 90% albumin binding for each ligand, we proceeded to apply the Serum Enhanced Binding (SEB) test to a separate group of patients: 12 diagnosed with cirrhosis and 12 with no liver dysfunction. It's important to note that these patients were different from those used in the analytical development of the SEB test.

In brief, independent solutions of Cu and Cd at 1190 µM, Au at 11,900 µM, and L-thyroxine at 75 µM were individually incubated with 200 µL of serum for Cd, Cu, L-thyroxine, and dansylsarcosine to achieve HSA/ligand theoretical ratios (mol/mol) of 1/5. For Au, 50 µL of serum was incubated to obtain an HSA/ligand ratio of 1/50. The same ligands and concentrations were also applied to rat plasma albumin in our animal models for comparison.

*ICP-MS analysis*: Calibration curves were constructed for each element using a six-point calibration range of 10 to 100 µg/L for Cu, Cd, Au, and sulfur, and 1 to 20 µg/L for L-thyroxine. Sulfur calibration employed L-cysteine, while iodine calibration utilized L-thyroxine. The KED mode was applied with oxygen at a flow rate of 0.3 ml/min for both calibrators, controls, and ultrafiltrates. When necessary, ultrafiltrates were diluted with 0.1 M HNO_3_. Cu was measured at m/z 65, Cd at m/z 112, Au at m/z 197, iodine at m/z 127, and sulfur at m/z 48, following previously described methods^19^. To standardize SEB test results across samples, the concentration of each ligand in the ultrafiltrate was normalized by the total albumin concentration in the sample.

### Animal experiments

All animal care and experimental procedures were approved by the French Ministry of Higher Education, Research and Innovation (APAFIS reference APAFIS#20,354–2,019,042,414,581,742) and were performed in accordance with the European Communities Council Directive guidelines for animal experimentation of the (EU/63/2010). These experiments and procedures are reported according to the ARRIVE guidelines^20,21^ with the recommendations made by French Ministry of Higher Education. All methods are reported in accordance with ARRIVE guidelines (https://arriveguidelines.org).

For all the included animals, weight was recorded daily throughout the experimental period until sacrifice. Animals were euthanised with intraperitoneal injection of pentobarbital (150 mg/kg). Blood was collected in Vacutainer® lithium heparin tubes (Beckton Dickinson, France) and centrifuged at 3000 rpm for 10 min. The resulting plasma samples were stored at -80 °C for analysis. The liver was promptly excised and fixed in formalin for histological analysis.

#### Induction of ethanol (EtOH) hepatotoxicity

Six different groups of 6 rats were orally administered (by gavage) 2 mL of a 50% EtOH solution (equivalent to 0.4 g or 1.6 g/kg of body weight) prepared in physiological saline (0.9% NaCl). The groups were followed for 1, 3, 7, 10, or 14 days, respectively to evaluate the time-dependent changes in biochemical markers and histological liver injuries. The animals were sacrificed 24 h after their last intake of EtOH. Control rats (n = 6) received 0.9% NaCl by gavage for 14 days.

#### Induction of carbon tetrachloride (CCl_4_) hepatotoxicity

Five groups of 6 rats were orally administered (by gavage) 1 mL/kg body weight of a 30% solution of CCl_4_ diluted in olive oil (equivalent to 1.594 g/kg of body weight). The groups were followed for 1, 3, 7, 10 days, respectively and the animals were sacrificed 24 h after their last intake of CCl_4_. Control rats (n = 6) received olive oil by gavage for 10 days.

#### Pathological analysis of the liver

After animal sacrifice, the liver was cut into sections of 1 to 1.5 cm perpendicular to the major axis to allow homogeneous fixation in a 4% formalin solution, and kept at ambient temperature for a maximum of 7 days. Samples were stained for light microscopy with hematoxylin, eosin and Masson's trichrome. The pathologist performed histological analysis blindly of the experimental groups.

#### Biochemistry analyzes

From the collected plasma, measurements of classic biochemistry parameters such as albumin (ALB), total (BILIT) and conjugated (BILID) bilirubin, aspartate aminotransferases (ASAT), alanine aminotransferases (ALAT) and alkaline phosphatases (PAL) were determined using a COBAS® 8000 system (Roche, Germany).

#### Characterization and quantification of albumin isoforms in rat plasma

Albumin isoforms were determined using the method described elsewhere^22^. Briefly, 20 µL of plasma were diluted with 980 µL of an aqueous solution of 20mM ammonium formate with 0.1% formic acid, vortex-mixed before filtration on a 0.22 µm cellulose filter and then injected on LC-HR-MS system (Nexera LC40 system coupled to a TripleTOF® 5600+, Shimadzu, Noisiel, France and Sciex, Concord, Canada). The LC-HRMS data were processed using PeakView® 2.2 and its Bio Tool Kit 2.2.0 (Sciex). The input MS spectra were filtered-in between 1300 to 1600 and then deconvoluted at low resolution (5000) between m/z 1000–200,000.

### Molecular dynamics simulations

The initial HMA model was obtained from the protein data bank (PDB ID 5GIX) which has been co-crystallized with seven palmitate (PLM) molecules^23^. Missing N- and C-terminal residues were added using the Modeller software ^24^. Protonation states of titratables residues (namely arginine, lysine, glutamate and aspartate) were determined using the H++ server assuming a physiological pH at 7.4. Since HMA was shown to be natively bound to PLM with a molecular ratio ranging from 0.1 to 2.0 in physiological conditions, we built the present HMA model with two PLM molecules, which were considered docked in fatty acid binding sites (FABS) 2 and 5^25,26^. The apo form of HMA was also considered in the present investigations. Three models of palmitate-bound PTM albumins were also built in the present study, namely HNA1, HNA2 and N-truncated HMA. All the systems were solvated in an explicit water box for which minimal distance between atoms and box edges were set up at 10 Å. Systems were all neutralized considering physiological NaCl salt concentration ([NaCl] = 154 mM). FF14SB^27^ and TIP3P^28,29^ forcefields were used to respectively model protein residues and water molecules. Parameters from Joung and Cheatham^30^ were used to model Na+ and Cl- counterions. Parameters for PLM, S-bonded cysteine were derived from Lipid17^31^ and FF14SB force fields while those for cysteic acid residues were derived from amber 99SB-based parameters available in the literature ^32^. Each replica was minimized and then equilibrated for 10.25 ns. MD production run was performed for 2 μs. Simulations were analyzed using the CPPTRAJ package^33^, and in house python scripts. Plots were obtained using the matplotlib v3.7.0 Python package^34^. Rendering was prepared using VMD software (alpha-v1.9.4). Structural clustering was carried out using density peak algorithms^35^, with inter-subdomain distances as metrics. Clusters representing more than 10% of the overall conformational space were considered. Allosteric communications were calculated with the Allopath Tool^36^.

### Declaration of transparency and scientific rigour

This study involved animal experimentation: This Declaration acknowledges that this paper adheres to the principles for transparent reporting and scientific rigour of preclinical research as stated in the scientific reports guidelines for Design and Analysis, and Animal Experimentation, and as recommended by funding agencies, publishers and other organisations engaged with supporting research.

### Ethics and integrity Statements

Declaration of transparency: this declaration acknowledges that this paper adheres to the principles for transparent reporting and scientific rigour of preclinical research as stated in the scientific reports guidelines for design and analysis, and as recommended by funding agencies, publishers and other organisations engaged with supporting research. All the methods were performed in accordance with the guidelines for animal experimentation of the European Communities Council Directive (EU/63/2010). The animal experiments and procedures are reported according to the ARRIVE guidelines (https://arriveguidelines.org).

## Results

### Enhanced binding capacity of serum/HSA

**Figure 1:**
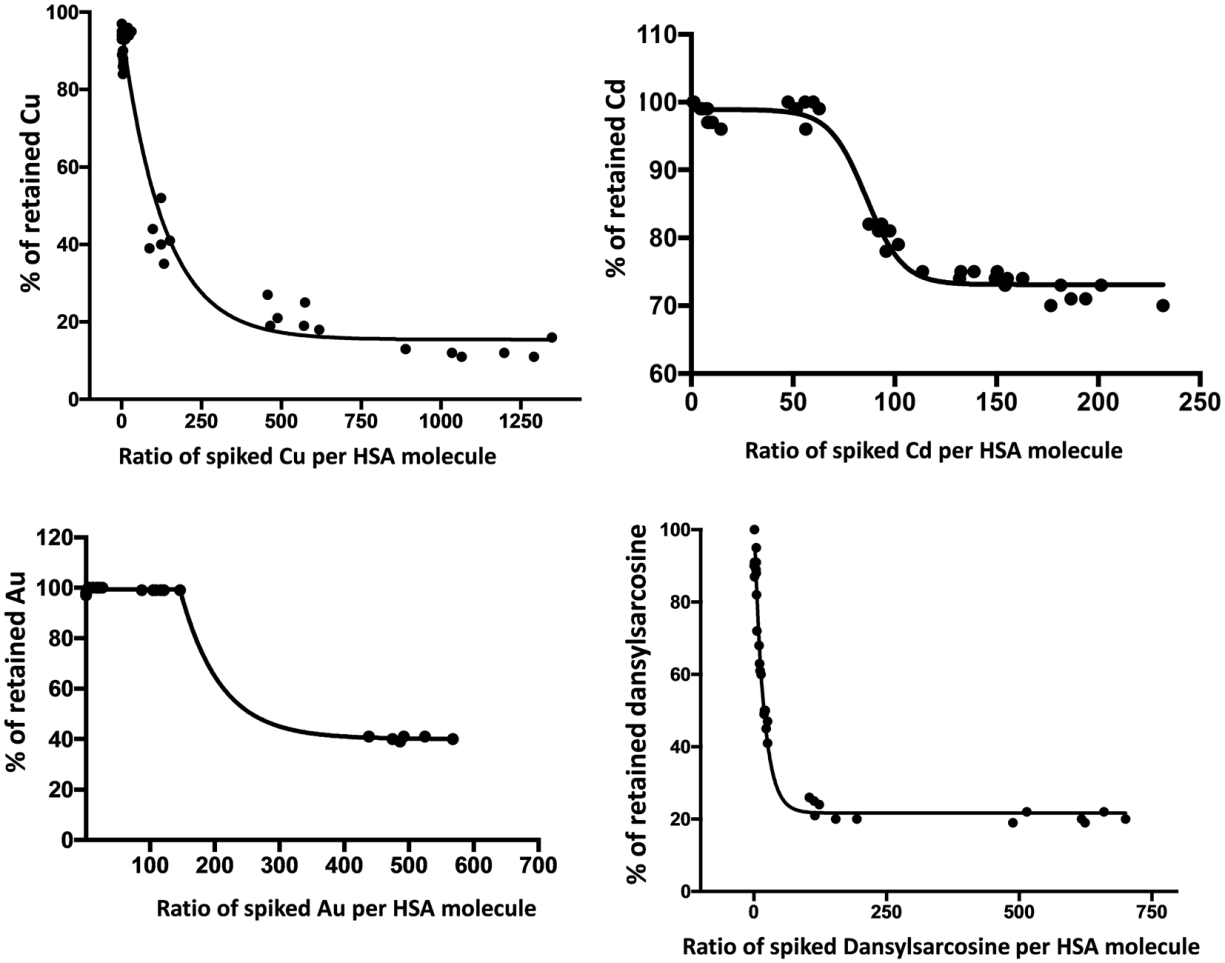
Serum enhanced binding of Cu, Cd, Au and dansylsarcosine.

By adding increasing concentrations of Cu to serum, we observed that up to 12 Cu atoms per albumin molecule were retained on the filter with an average retention of 95%. This percentage dropped to 40% or less when more Cu was added (Fig. 1). Serum samples were able to bind up to 150 atoms of Au, 50 atoms of Cd, 2.5 molecules of dansylsarcosine and 10 molecules of L-thyroxine per molecule of albumin, all with near 100% retention. However, L-thyroxine could not be tested further than 1/10 (HSA/L-thyroxine) because of dissolution problems. The binding capacities of the commercial solution of pure HSA Vialebex® at 200 mg/mL (40 Cu per HSA and 150 Au per HAS for example) were equivalent or higher than those of patient serum samples (data not shown), suggesting that binding to other proteins is negligible.

### Comparison of HSA binding capacities in patients with liver cirrhosis or no hepatic dysfunction

**Figure 2:**
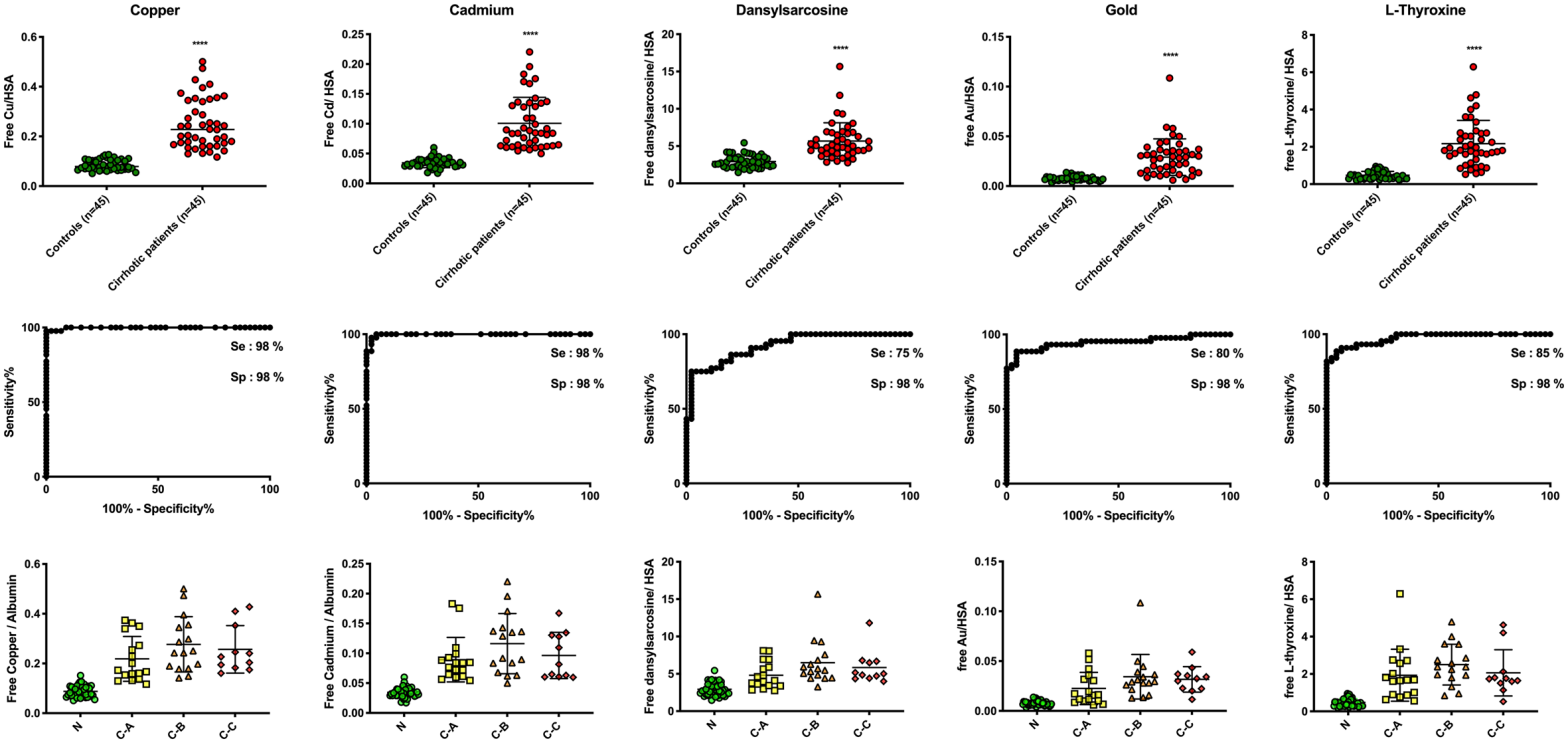
SEB test performance to discriminate cirrhosis patients from control patients.

**Figure 3:**
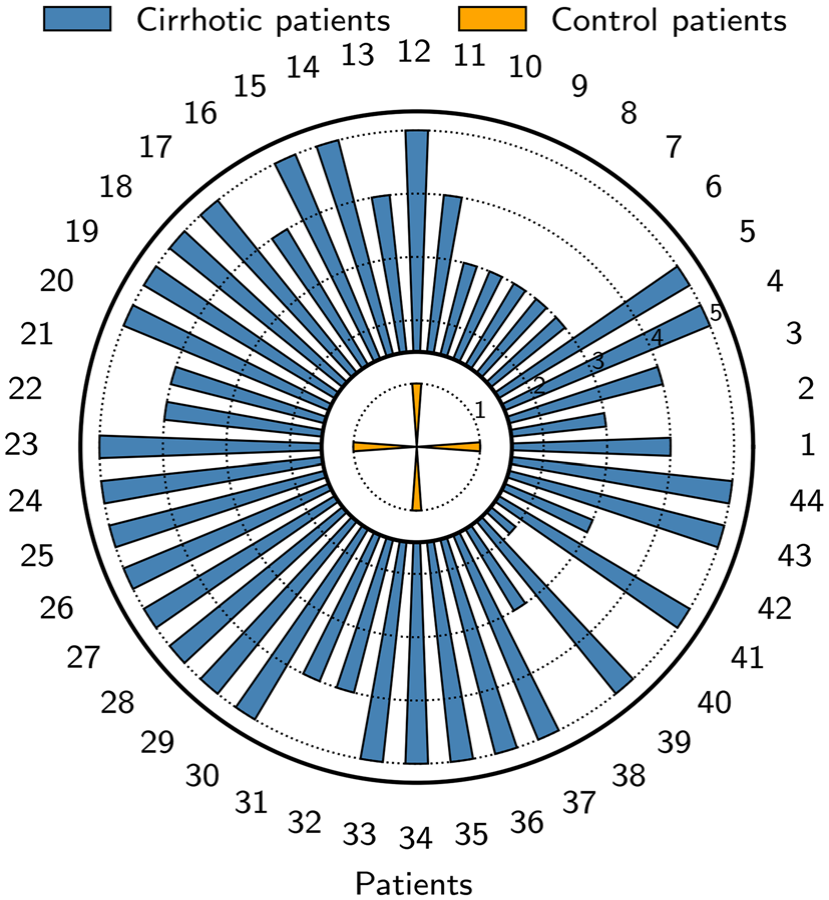
Number of positive ligands of the SEB test for each patient included in the cohort.

Among the 45 cirrhotic patients, 17 (37.8%) were diagnosed with Child–Pugh score A, 16 patients (35.5%) Child-Plugh B and 10 patients (22.2%) Child–Pugh C. The ligands of the SEB test had individually excellent sensitivity and specificity to discriminate cirrhotic patients from control patients (Fig. 2). All the cirrhotic patients had at least 3 ligands above threshold (except one patient who had only 2) (Fig. 3): 26 patients (57.8%) had 5 ligands above threshold, 9 patients (20%) had 4 and only 7 patients (15.5%) had 3 positive ligands. Among the 45 control patients, only 4 patients had one ligand above threshold. None of the ligands alone was able to discriminate patients regarding their Child–Pugh score (Fig. 2).

### Early diagnostic performance of the SEB test in animal models

**Figure 4:**
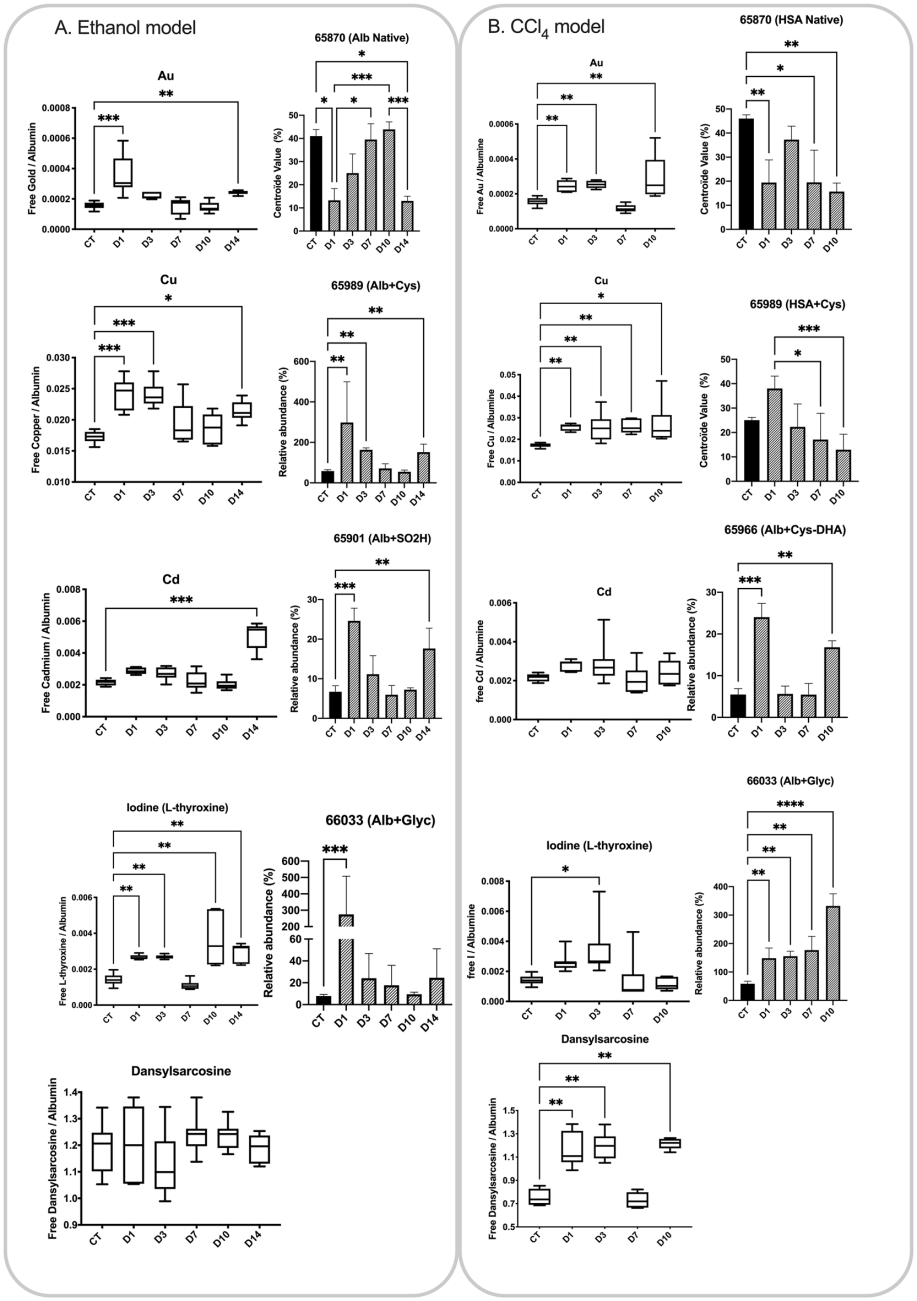
SEB test ligands evolution and albumin isoforms in the rat models. (**A**) Ethanol model. (**B**) CCl_4_ model.

After daily administration of 1.6 g ethanol/kg or 1.59 g CCl_4_/kg of body weight to different rat groups, the SEB test was performed in the serum of all the rats using Cu, Cd, L-thyroxine, dansylsarcosine at 1:5 mol ratio and Au at 1:50 mol ratio as described above. In the EtOH model, on D14, all the rats were positive for all the ligands except dansylsarcosine (Fig. 4A). The Au binding capacity was decreased on D1 (24h after the first dose), restored on D7 and reduced again thereafter. The same behavior was observed for the binding capacity of Cu and L-thyroxine. In contrast, the binding capacity of Cd was only decreased on D14 (Fig. 4B). In the CCl_4_ model, albumin-binding capacities were reduced for Cu, Au and dansylsarcosine (except on D7), but not for Cd at any time (Fig. 4B).

In both models, the decrease of albumin-binding was paralleled by albumin modifications. Native albumin was decreased as soon as 24h after the administration of hepatotoxic compound. It then increased back at D3 and D7 in the CCl_4_ and EtOH models, respectively, prior to a final drop observed at D10 or D14. Decreases in native albumin were associated with increases of ALB isoforms, as shown in Fig. 1 (e.g., ALB + Cys, ALB + SO_2_H or glycated albumin, see Fig. 4A,B).

**Figure 5:**
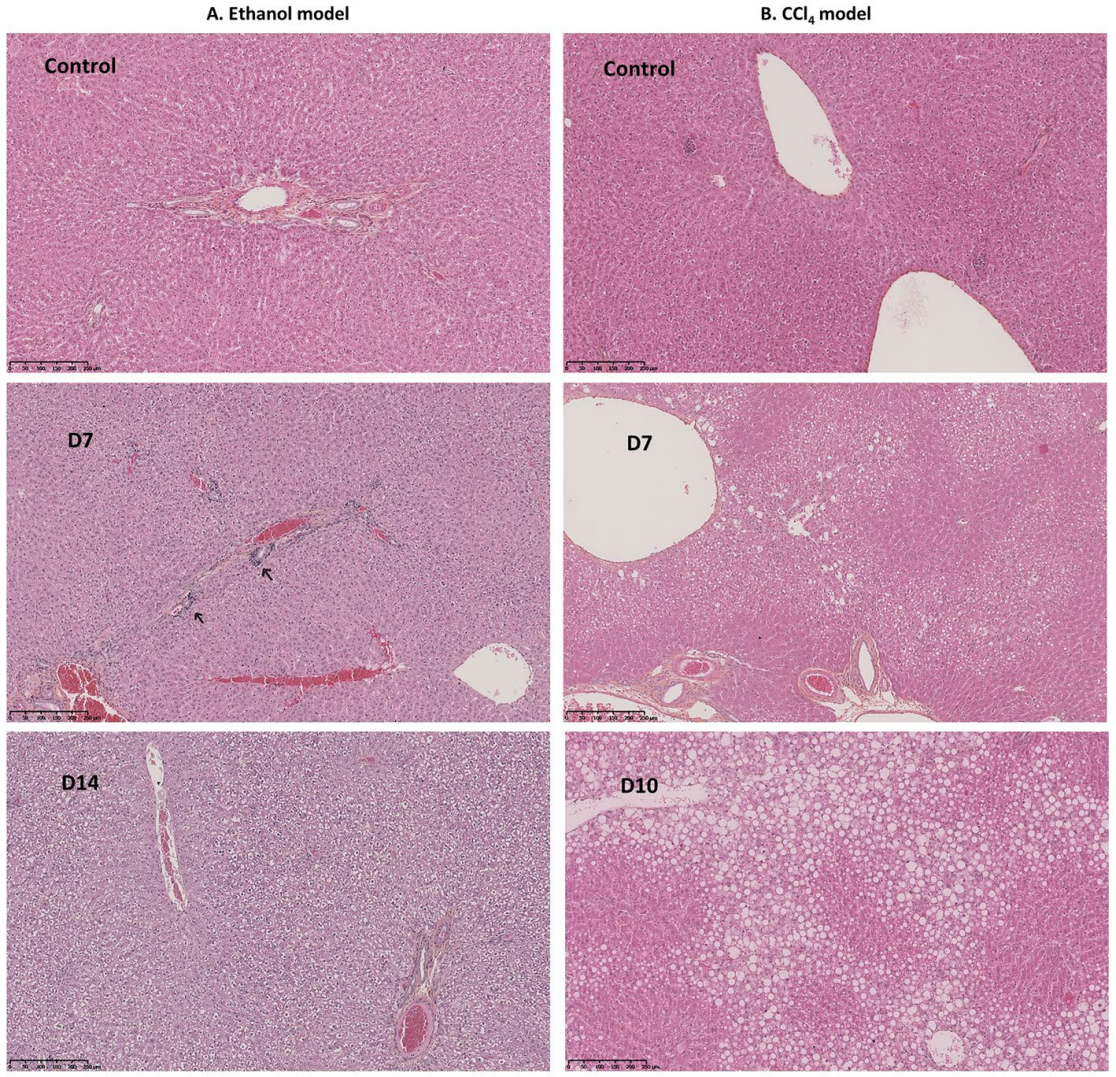
Comparative histopathological analysis of liver sections in ethanol and CCl_4_-induced liver injury models. Panel A shows liver sections from an ethanol model and Panel B from a CCl_4_ model, with control, Day 7 (D7), and Day 14 (D10) time points. Hematoxylin and eosin staining; scale bars = 100 µm.

In the EthOH model, a significant increase in AST level was observed at D7, D10 and D14 (Table 1). In the CCl_4_ model, AST and ALT levels were significantly increased at all time points and bilirubin (BILIT & BILID) levels at D7 and D10 (Table 2). Histologically, only minor inflammation was detected in some rats receiving ethanol. Rats exposed to CCl_4_ exhibited steatosis from D3 onwards and 2 rats exhibited fibrotic liver tissue (1 on D3 and 1 on D10) (Fig. 5).

**Table 1:** Effect of EtOH on biochemical markers expressed as the median [min–max] (*p < 0.05).

Biomarkers	Controls	D1	D3	D7	D10	D14
ALB (g/L)	14.4 [12.7–17.6]	13.8 [13.2–14.1]	12.4* [11.8–14.2]	12.2* [11.0–13.8]	16.35 [12.4–17.7]	12.9* [10.2–13.5]
AST (UI/L)	70.8 [61–75]	78 [70–85]	80 [52–144]	96* [75–157]	92* [81–157]	90* [85–294]
ALT (UI/L)	57 [46–61]	63 [52–71]	62 [46–123]	69 [52–97]	78* [68–98]	82* [65–104]
PAL (UI/L)	196 [101–325]	232 [153–304]	180 [121–329]	159 [84–263]	172 [100–222]	230 [110–327]
BILID (µM)	0.75 [0.6–1]	0.7 [0.3–1]	0.7 [0.5–1.1]	0.7 [0.4–4.4]	0.8 [0.5–1.1]	0.6 [0.4–0.7]
BILIT (µM)	0.75 [0.4–1.3]	0.8 [0.4–1.6]	0.8 [0.2–1.7]	1.0 [0.2–1.9]	1.0 [0.8–1.6]	0.9 [0.5–1.8]
Histology		2/7 Inflammation	1/6 inflammation	4/7 inflammation	2/8 inflammation	3/9 inflammation

**Table 2:** Effect of CCl_4_ administration on biochemical markers expressed as the median [min–max] (* p < 0.05; ** p < 0.01).

Biomarkers	Controls	D1	D3	D7	D10
ALB (g/L)	**14.4** **[12.7–17.6]**	**14.2** **[12.9–14.5]**	13.0* **[11.8–14.2]**	12.9* **[10.4–14.5]**	**16.0** **[15.9–16.7]**
AST (UI/L)	70.8 [61–75]	123* **[90–251]**	151* **[82–214]**	232** **[168–305]**	337** **[192–473]**
ALT (UI/L)	**57** **[46–61]**	77* **[67–181]**	90* **[78–208]**	200** **[107–638]**	355** **[166–538]**
PAL (UI/L)	196 [101–325]	232 [192–270]	237 [174–329]	248 [117–341]	205 [149–250]
BILID (µM)	**0.75** **[0.6–1.0]**	**1.1** **[0.7–1.5]**	**1.2** **[0.3–1.3]**	1.6** **[1.2–2.0]**	2.5** **[1.8–2.9]**
BILIT (µM)	0.75 **[0.4–1.3]**	1.4 [0.4–2.0]	1.3 **[0.6–3.5]**	2.3** **[1.8–4.5]**	3.5** **[2.4–5.3]**
Histology			Steatosis 5/5 1/5 fibrosis	Steatosis 6/6	6/6 steatosis 1/6 fibrosis

### Structural variabilities of HSA isoforms

**Figure 6:**
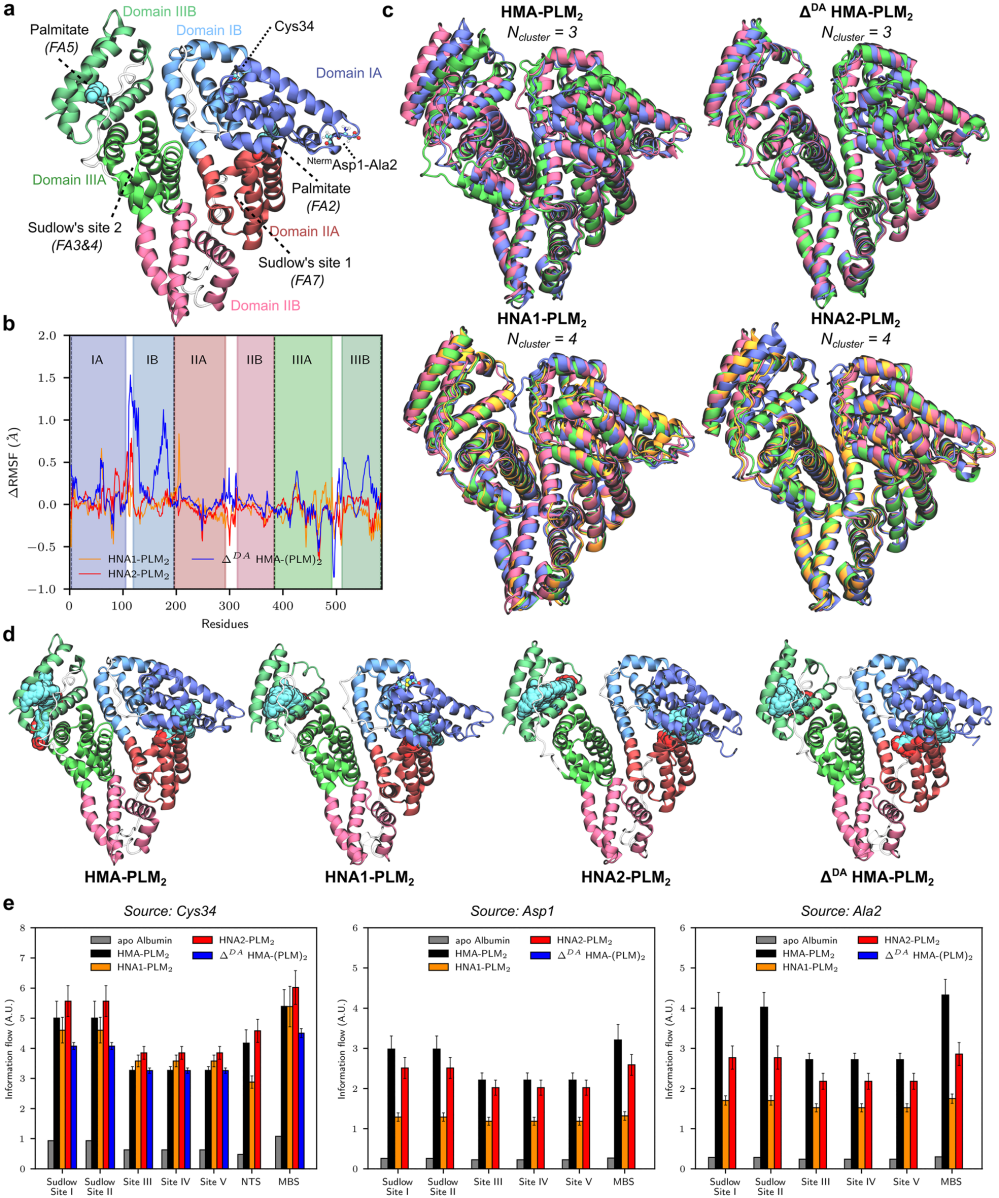
Structural and dynamic variabilities of HSA isoforms. (**a**) Overview of HMA albumin used as reference. Domains I, II and III are coloured blue-ish, red-ish and green-ish, respectively, for which each subdomain A and B are stressed out. Residues modified by PTM considered in this work are also located, namely N-terminal Asp1 and Ala2 residues for Δ^DA^ HMA as well as Cys34 for HNA1 and HNA2. Sudlow’s sites 1 and 2 are also shown as well as the two main fatty acid binding sites used in the present models (FA2 and FA5). (**b**) Per-residue Root Mean Squared Fluctuation differences (ΔRMSF, Å) between PTM HSA isoforms and native HMA. Subdomains are shown as background. (**c**) Representative snapshots of each structural cluster obtained from MD simulations for each HSA isoform. (**d**) Structural variabilities of bound palmitates to HSA isoform representative snaphots. 300 different palmitate molecules along MD trajectories are displayed for FA2 and FA5. (**e**) Allosteric communication between different sources (Cys34, Asp1 and Ala2) to main albumin binding sites, namely Sudlow’s site 1 and 2 and L-thyroxine site 3, 4 and 5, as well as metal binding sites (N-terminal binding site–NTS—and Metal binding site–MBS). Efficiency of information flow from source to sink is shown from current flow closeness from the Allopath tool.

The secondary structure of albumin was not affected by Cys34 oxidation nor N-truncation as pictured by the backbone root-mean square deviations (RMSD) versus X-ray structure^23^ ranging from ca 3.0 and 5.0 Å (Supplementary Figure S1-S4). MD simulations revealed that albumin isoforms adopted different conformational dynamics of inter-domain arrangements. Inter-subdomain distances were monitored showing the impact from Cys34 oxidation or N-truncation (Supplementary Figure S5). This is particularly true for the following subdomain pairs: IA-IIIB, IB-IIIB IIA-IIIB and to a lesser extent IA-IIA, IA-IIB IIA-IIIA. Representative structures were obtained from structural clustering and are shown in Fig. 6. Palmitate-bound HMA and HNA2 isoform exhibited similar patterns in which the overall structure showed dynamic closing of central cleft defined by the distance between domains I and III for which domain II acts as pivot. Palmitate-bound HNA1 and Δ^DA^ HMA isoforms showed less variability in terms of domain arrangements. Palmitate-bound HNA1 clusters are slightly more open cleft conformations, The N-truncated Δ^DA^ HMA isoform only populated open-cleft conformations pictured by larger distances between subdomain IA/IB and IIIB (Fig. 5c and Supplementary Figure S5). This was confirmed by the largest RMSDs when comparing Δ^DA^ HMA subpopulations with other isoforms (Supplementary Figure S6).

Subtle differences in term of structural dynamics were observed by comparing per-residue root-mean-squared fluctuations with those of native HMA (ΔRMSF, Fig. 5b). Simulations revealed larger deviations for domains I and III. This is particularly true for the domain I of Δ^DA^ HMA isoforms which exhibited ΔRMSF up to 1.5 Å. To lesser extent, HNA1 and HNA2 also exhibited similar trends.

Albumin PTMs leads to subtle but significant differences which may be associated with different binding capacities. Different dynamics for the bound palmitate molecules were observed. The FA5 palmitate molecule showed larger structural variability for HMA than for other isoforms as shown by different binding modes for during MD simulations. In contrast, the FA5 palmitate molecule remains tightly bound for HNA1, HNA2 and Δ^DA^ HMA isoforms. It may suggest a lower binding affinity for HMA which might favour palmitate substitution by other ligands. Interestingly, no significant difference regarding palmitate dynamics in FA2 was observed. This might be explained by (i) the known highest affinity of FA2^25,37^ and (ii) its location in domain IIA which was not shown to be affected by PTMs.

Albumin PTMs might affect the distant communications between the different subdomains. To this end, the efficiency of the information flow from Cys34 or Asp1-Ala2 sequence to different substrate binding sites were assessed for every systems (Fig. 1e). Allosteric communications from Cys34 to all binding sites was is significantly lower in the N-truncated Δ^DA^ HMA isoform than in others. Likewise, HNA1 and HNA2 isoforms exhibited lower allosteric communications from N-terminal region to all the binding sites. Oxidation of Cys34 might disrupt the propagation of the information flow from N-terminal domain to the rest of the protein. Finally, metal binding capacities were assessed by simply considering electrostatic potentials for each representative snapshots (Supplementary Figure S7). Open-cleft conformations lead to more exposed electropositive regions which may be associated with larger repulsion with cationic metals. This may be relevant for the N-truncated Δ^DA^ HMA isoform for which only such a population was observed during MD simulations.

## Discussion

We developed a multi-element functional test of albumin binding capacities, whose results parallel the relative decrease in native human serum albumin and discriminate patients with liver cirrhosis from controls. When applied to two different induced hepatotoxicity models in rats, the SEB test detected liver injuries very early, when the most important posttranslational modifications of albumin also appeared, contrary to classical functional liver tests. Notably, the ethanol-induced hepatotoxicity model exhibited more favorable results in terms of early liver damage detection compared to the CCl_4_ model.

The ligands used for the SEB test were carefully selected based on their binding sites to albumin in order to cover the most important albumin modifications that may occur in liver dysfunctions. This was inspired by recent studies by Baldassare et al. and Dominicali et al*.* showing the decrease of effective albumin (the native form) and the increase of several isoforms in advanced liver diseases^2,4^. Three metals, Au, Cu and Cd, were selected to respectively monitor modifications occurring at (i) the Cys34 position^37–39^, (ii) the N-terminal site and the multi binding site B^39^ and (iii) multi binding sites A (also known as Cd binding site)^12^. Organic ligands were also considered. L-thyroxine was shown to possibly bind up to 5 binding sites^40^ and dansylsarcosine having a binding site located in the drug site2^6^. Albumin binding capacities toward dansylsarcosine and L-thyroxine are expected to reflect conformational modifications since their binding sites are positioned in the cavities of the protein. It is worth mentioning that the ligands used to perform the SEB test can be directly measured using a single and rather straightforward analytical method based on inductively coupled plasma mass spectrometry (ICP-MS), and possibly by means of other analytical procedures. Our analytical approach is in line with a former work by Bar-Or et al*.* in the context of cardiac ischemia. They proposed the albumin-cobalt binding test (ACB), also known as the Ischemia Modified Albumin test (IMA)^41^. The IMA test is based on the hypothesis that cardiac ischemia is associated with modifications of albumin structure which, in turn, decrease cobalt binding. Briefly, the IMA test is performed by adding CoCl_2_ and dithiothreitol to serum, followed by a colorimetric measurement of the (free-Co)-dithiothreitol complex at 470 nm. Although there is some controversy about the origin of the decreased HSA capacity to bind cobalt, a correlation between the albumin binding functions and the severity of cirrhosis was observed using the same test ^7^. Importantly, in these applications, given that albumin represents 60% of serum proteins, it is assumed that any significant decrease of HSA binding capacity for a ligand with a high affinity is prominently due to a modification on its albumin-biding site.

In developing the SEB test, we determined the maximum binding capacities of different ligands. Serum can bind up to 150 atoms of Au, 50 atoms of Cd, 50 atoms of Cu, 2.5 molecules of dansylsarcosine, and at least 10 molecules of L-thyroxine per albumin molecule (Fig. 1). Our study goes beyond assessing known albumin binding sites by using high ligand concentrations to investigate adsorption on the albumin surface (except for dansylsarcosine). Molecular modeling shows that PTMs affect albumin dynamics and inter-domain communications, particularly with Cys34 oxidation or N-truncation. Dansylsarcosine predominantly binds Sudlow's site 1, minimally affected by PTMs, but PTMs impact Sudlow's site 2 and FA binding site 5 more in HNA1 and ΔDA albumin isoforms. This may explain lower binding affinity for a second dansylsarcosine binding.

In the preliminary phase, various molar ratios (ligands/albumin) were tested on patients with liver diseases and control patients. Surprisingly, even at molar ratios below maximum binding capacities, all the ligands effectively differentiated cirrhotic from non-cirrhotic patients with satisfactory sensitivities and specificities (data not shown). Based on these findings, we selected the lowest effective ratios observed in patients for further application in a cohort of cirrhotic and control patients, as well as in our animal experiments. The SEB ligands successfully discriminated cirrhotic patients, regardless of the Child–Pugh score, further supporting our hypothesis that albumin PTMs occur early during liver injury. However, it is important to acknowledge a potential limitation of our study, as we relied on an animal model to test this hypothesis due to the absence of crystalized rat albumin. Nevertheless, the results obtained in our study align with the assumption that the same binding sites and mechanisms apply to rat albumin, reinforcing the validity of our findings.

In the animal hepatotoxicity models, the decrease in the binding capacities of the SEB test ligands was correlated with the decrease of the native albumin fraction to the benefit of the other isoforms. Although the decrease of native albumin might explain the behaviors of some ligands as observed in Fig. 4, it is worth further interpretation. First, albumin oxidation on Cys34 resulted in the disruption of Au binding, leading to decreased Au-binding capacities in intoxicated rats. Cys34 oxidation was also associated to conformational and dynamic alterations of human albumin^6^, at the interface between domain I and domain I-II, leading to lower binding capacities of endogenous (L-tryptophan) and exogenous ligands (cefazoline and verapamil). The corresponding binding sites being distant from Cys34, allosteric modulation might explain lower binding capacity of Cd in some of our experiments. Actually, the Cd binding site has not been fully characterized yet, but would be in the vicinity of the L-tryptophan, cefazoline and verapamil binding site. Secondly, the normal binding capacities for Au, Cu and dansylsarcosine observed at D3 and/or D7 in the EtOH and CCl_4_ models is consistent with the normal levels of native albumin observed. This is likely due to a short phase of liver regeneration, which has been extensively reported at least for animals intoxicated with CCl_4_^42–44^. Thirdly, Cu is reported to have strong affinity for the N-terminal binding site and the metal binding site B (MBSB). However, we did not identify any isoform with a truncation of the N-terminal moiety, suggesting that MBSB modifications alone are responsible for decreased Cu binding. Also, we detected N-Glycation and hypothesized that they occurred on Lys199, Lys281, Lys439, and Lys525^8^, all located near the L-thyroxine sites. These modifications would alter local dynamics and in turn affect the L-thyroxin binding capacity. Finally, Cu binding capacity showed the earliest decrease in our models. Therefore, Cu could be considered as a very early sensitive marker of an albumin PTM. In contrast, decreased Cd binding capacity was observed only at later stages, suggesting that it could be used as a biomarker of more intense, or different, albumin PTMs.

Our results show that the use of one ligand (as proposed with the IMA test) might not be sufficient to detect albumin PTMs in general. Actually, the coverage of a larger part of the albumin structure seems to be key to detect liver injuries early. Further investigations including molecular modeling should be further performed to comprehend the albumin isoforms-ligands binding relationships and to finely tune the SEB test. It is noteworthy that, in our study, control patients were patients hospitalized in various departments within our hospital and included patients with other underlying diseases. Despite this heterogeneity, the SEB test ligands collectively allowed us to distinguish all liver disease patients. This strongly suggests that when considered together, these ligands exhibit specificity for liver injuries.

The SEB test and albumin PTMs show potential for early detection of Drug-induced liver injury (DILI), a life-threatening adverse effect of certain drugs and a significant challenge in patient care and drug development. DILI is a major concern for regulators and the pharmaceutical industry, often leading to termination of drug development or post-approval withdrawal. Despite efforts to identify biomarkers, DILI diagnosis remains challenging due to the diverse presentations and lack of early, specific indicators. While some mechanistic biomarkers have shown promise, the extensive number of candidates highlights the unmet medical needs in DILI diagnosis and prediction. Timely medical care and prediction of outcomes, particularly in DILI-induced acute liver failure (ALF), are crucial. Prognostic biomarkers for overall DILI and predicting injury development in patients with normal-to-low ALT values are still lacking. Although micro-RNA-122 is the most promising biomarker to date, its clinical use is limited due to biological variations. The SEB test and albumin PTM monitoring hold promise in addressing these gaps, offering a patient-specific, temporal approach for DILI diagnosis and prognosis, despite potential challenges related to the price and limited availability of ICP-M in clinical laboratories. Future investigations are warranted to explore their broader potential in hepatology.

The primary limitations of our study are related to the fact that DILI are predominantly characterized by a dose-independent, idiosyncratic reaction, reflecting immunological rejection process rather than direct hepatotoxicity. Thus, our animal models may not perfectly replicate all facets of idiosyncratic liver injury but our study can still provide valuable insights into potential hepatotoxic effects and guide further experimental investigations. CCl_4_ and ethanol are more in line with a direct toxic effect rather than the idiosyncratic nature seen with some drugs, allowing us to study mechanisms of toxicity and potential protective interventions, which in our case are the SEB test and albumin isoforms. It is worth noting that the findings derived from cirrhotic patients in our research should not be directly extrapolated to the early detection of liver injury or DILI. Instead, they are utilized in this context to validate the efficacy of the SEB test^45^. Despite this, the early detection capability of the SEB test suggests its potential as a prognostic biomarker, including for DILI, pending further confirmation through future studies.

### Supplementary Information

Supplementary Information.

## Data Availability

The datasets used and/or analysed during the current study available from the corresponding author on reasonable request.

## Abbreviations

PTM: Post-translational modification
HAS: Human serum albumin
IMA: Ischemia Modified Albumin test
ABC: Albumin cobalt binding test
HMA: Human mercaptalbumin
HNA1: Nonmercaptalbumin 1
HNA2: Nonmercaptalbumin 2
Au: Gold
Cu: Copper
Cd: Cadmium
SEB: Serum enhanced binding test
CCl_4_: Carbon tetrachloride
ALP: Alkaline phosphatases
GGT: Gamma-glutamyl transferases
BILIT and BILID: Total and conjugated bilirubin
LDH: Lactate dehydrogenase
EtOH: Ethanol
FABS: Fatty acid binding sites
PLM: Palmitate molecules
RMSD: Root-mean square deviations
