# Supplementary material for: Early detection of liver injuries by the Serum enhanced binding test sensitive to albumin post-transcriptional modifications
Source: Sci Rep. 2024 Jan 16;14:1434. doi: 10.1038/s41598-024-51412-0 (PMC10791642; doi:10.1038/s41598-024-51412-0)
Supplement: Supplementary file 1 — Supplementary Information. [file 41598_2024_51412_MOESM1_ESM.docx]

**Early detection of liver injuries by the Serum enhanced binding test sensitive to albumin post-transcriptional modifications**

Souleiman El Balkhi^1,2^, Mohamad Ali Rahali^1,2^, Roy Lakis^1,^, François Ludovic Sauvage^1^, Marving Martin^1^, Angelika Janaszkiewicz^1^, Roland Lawson^1^, Ruben Goncalves^1^, Paul Carrier^1,3^, Veronique Loustaud-Ratti^1,3^, Anne Guyot^4^, Pierre Marquet^1,2^, Florent Di Meo^1^, Franck Saint-Marcoux^1,2^

**Author’s institutional affiliations:**

^1^ P&T, UMR1248, Inserm, Univ. Limoges, France.

^2^ Department of pharmacology, toxicology and pharmacovigilance, CHU Limoges, France.

^3^ Department of Liver Disease, CHU Limoges, Limoges, France

^4^ Department of Pathology, CHU Limoges, Limoges, France.

**Declarations of interest: none**

**Address for correspondence:** Souleiman El Balkhi, email: souleiman.elbalkhi@chu-limoges.fr


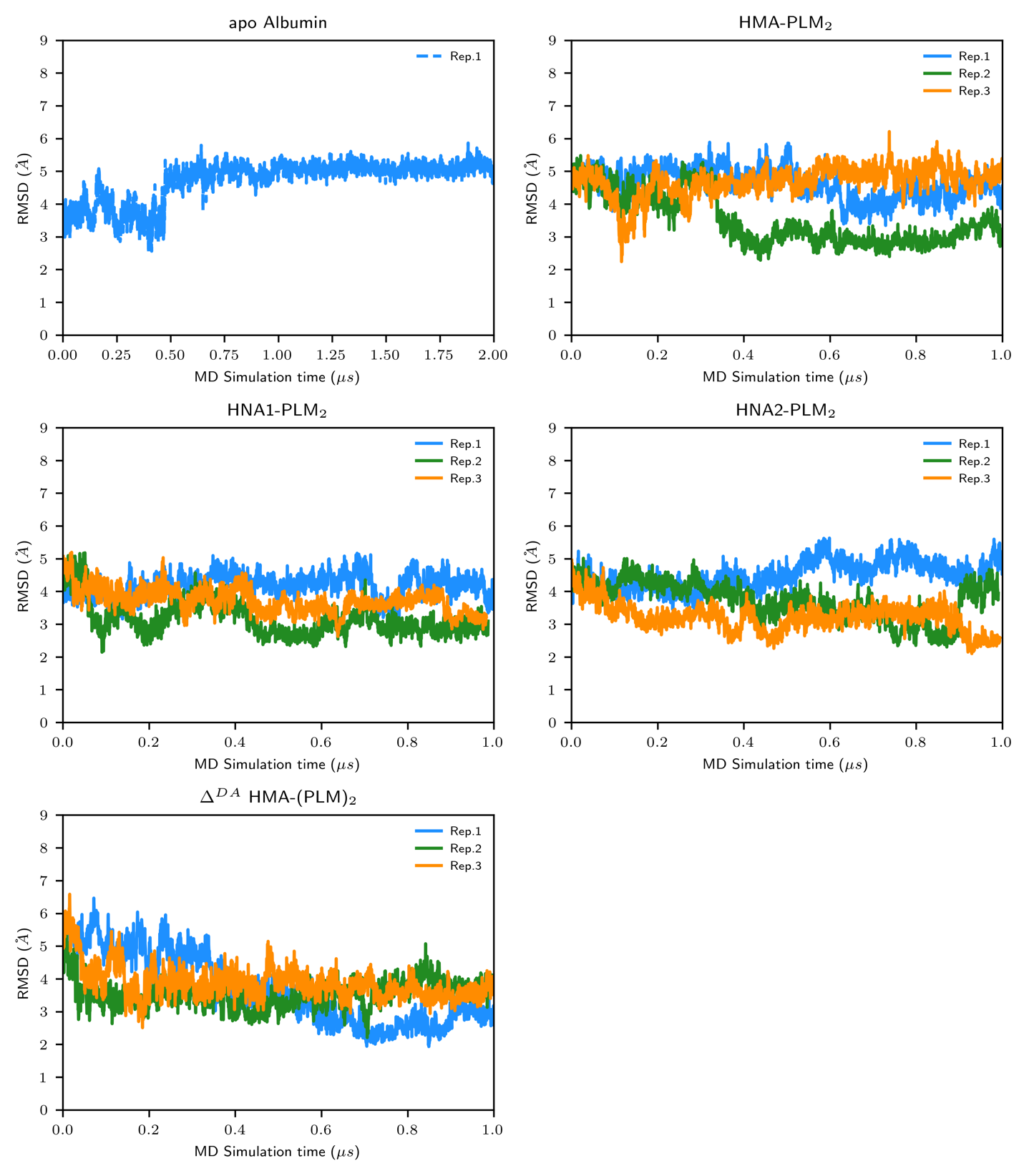


Supplementary Figure S1. Per-replica time-dependent root-mean squared deviations of overall backbone HSA isoform structures along MD simulations. The three different replicas are depicted in blue, green and orange, respectively.


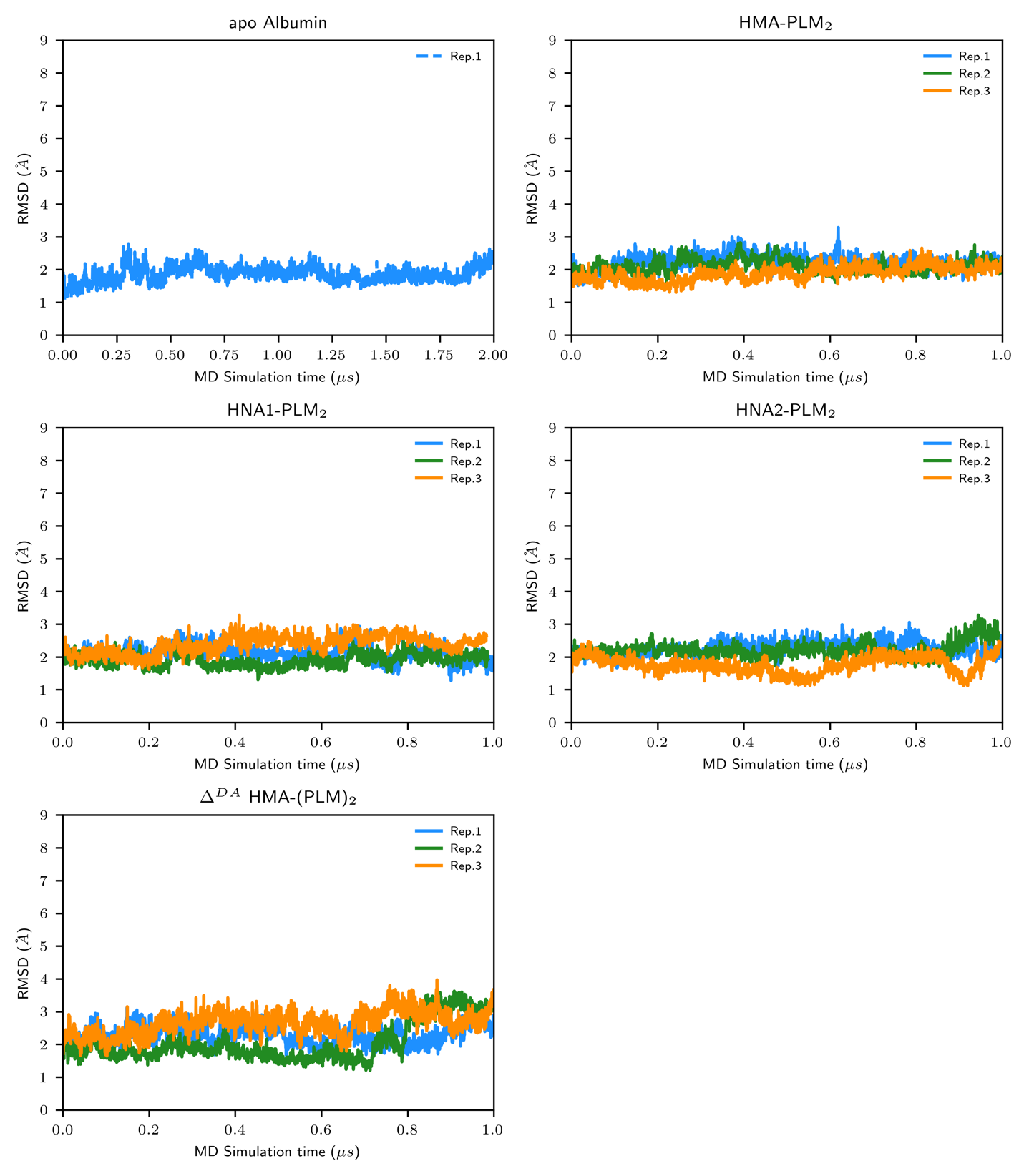


Supplementary Figure S2. Per-replica time-dependent root-mean squared deviations of backbone HSA isoform structures for domain I along MD simulations. The three different replicas are depicted in blue, green and orange, respectively.


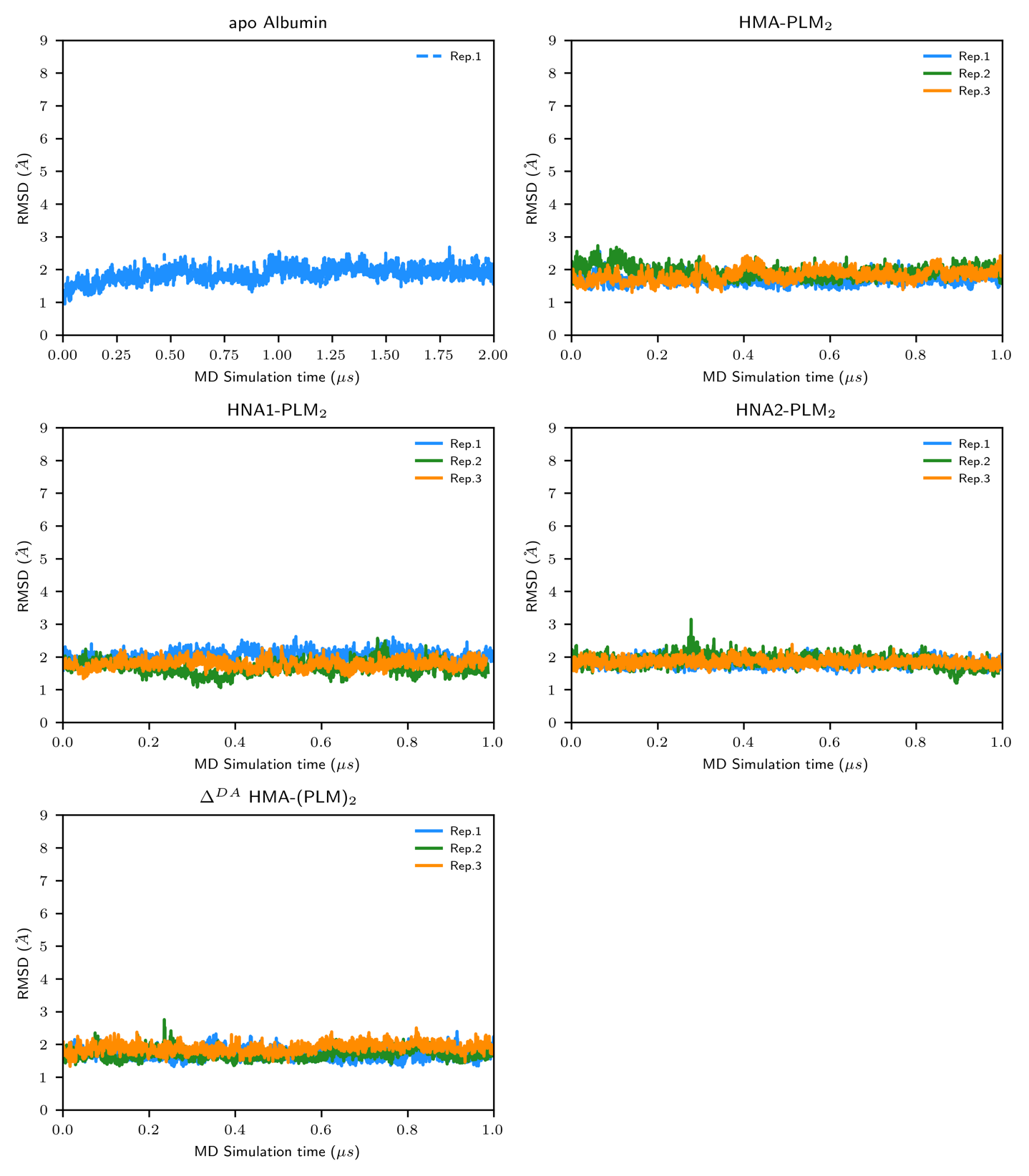


Supplementary Figure S3. Per-replica time-dependent root-mean squared deviations of backbone HSA isoform structures for domain II along MD simulations. The three different replicas are depicted in blue, green and orange, respectively.


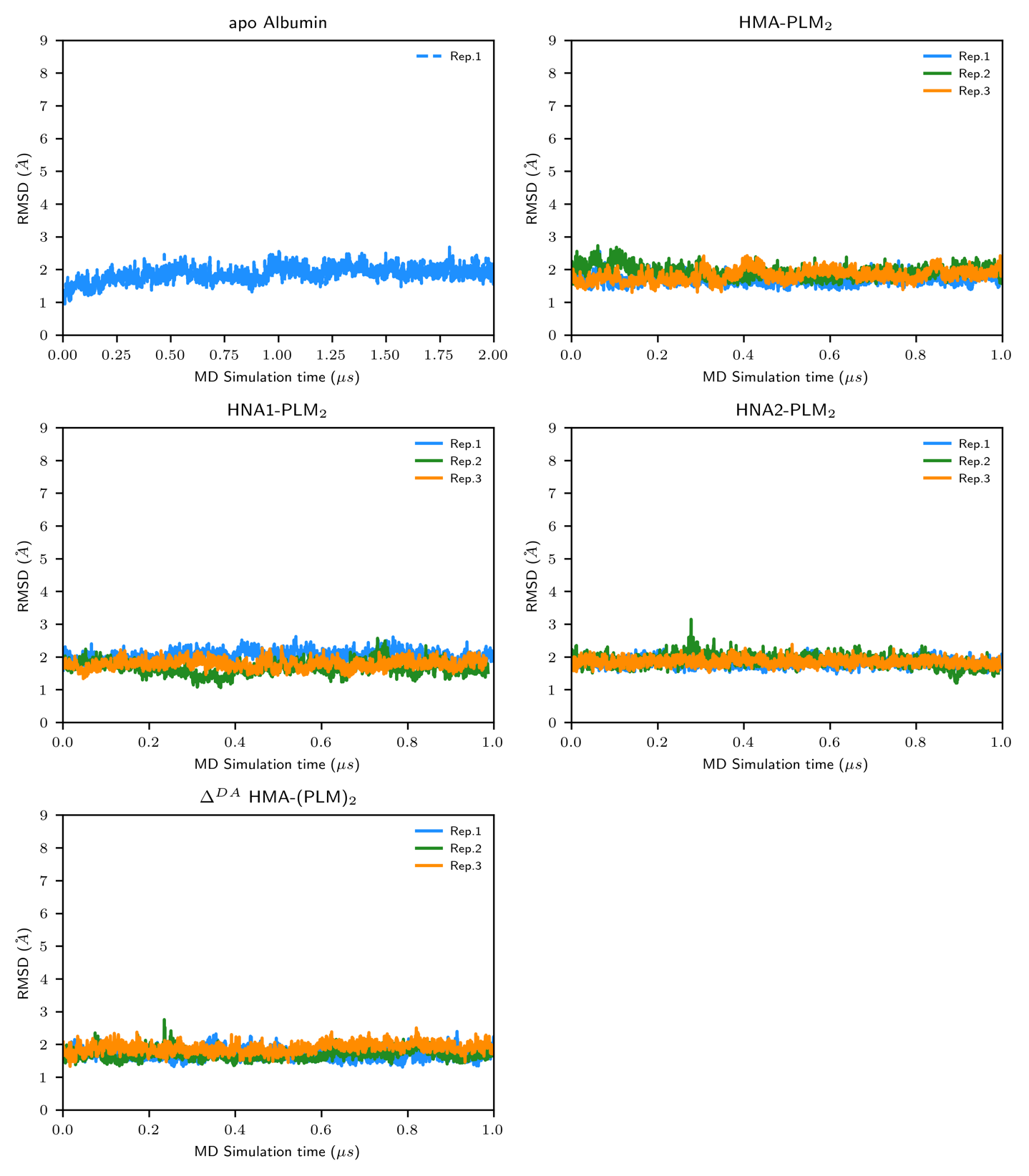


Supplementary Figure S4. Per-replica time-dependent root-mean squared deviations of backbone HSA isoform structures for domain III along MD simulations. The three different replicas are depicted in blue, green and orange, respectively.


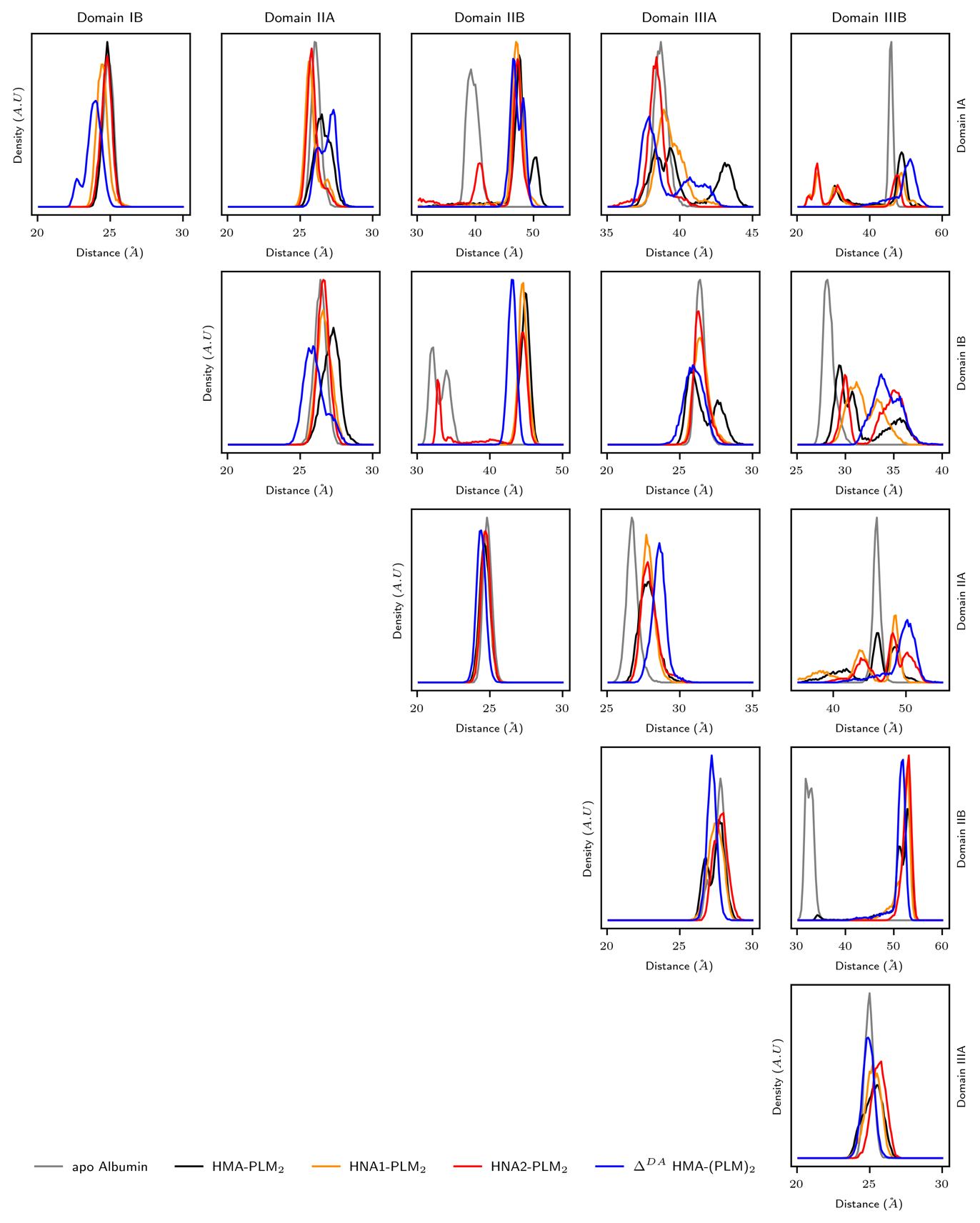


Supplementary Figure S5. Distributions of inter-subdomain distances (Å) for the different HSA isoform structures for which replicas were aggregated. For sake of comparison, distributions from MD simulation performed with apo HMA albumin were also plotted.


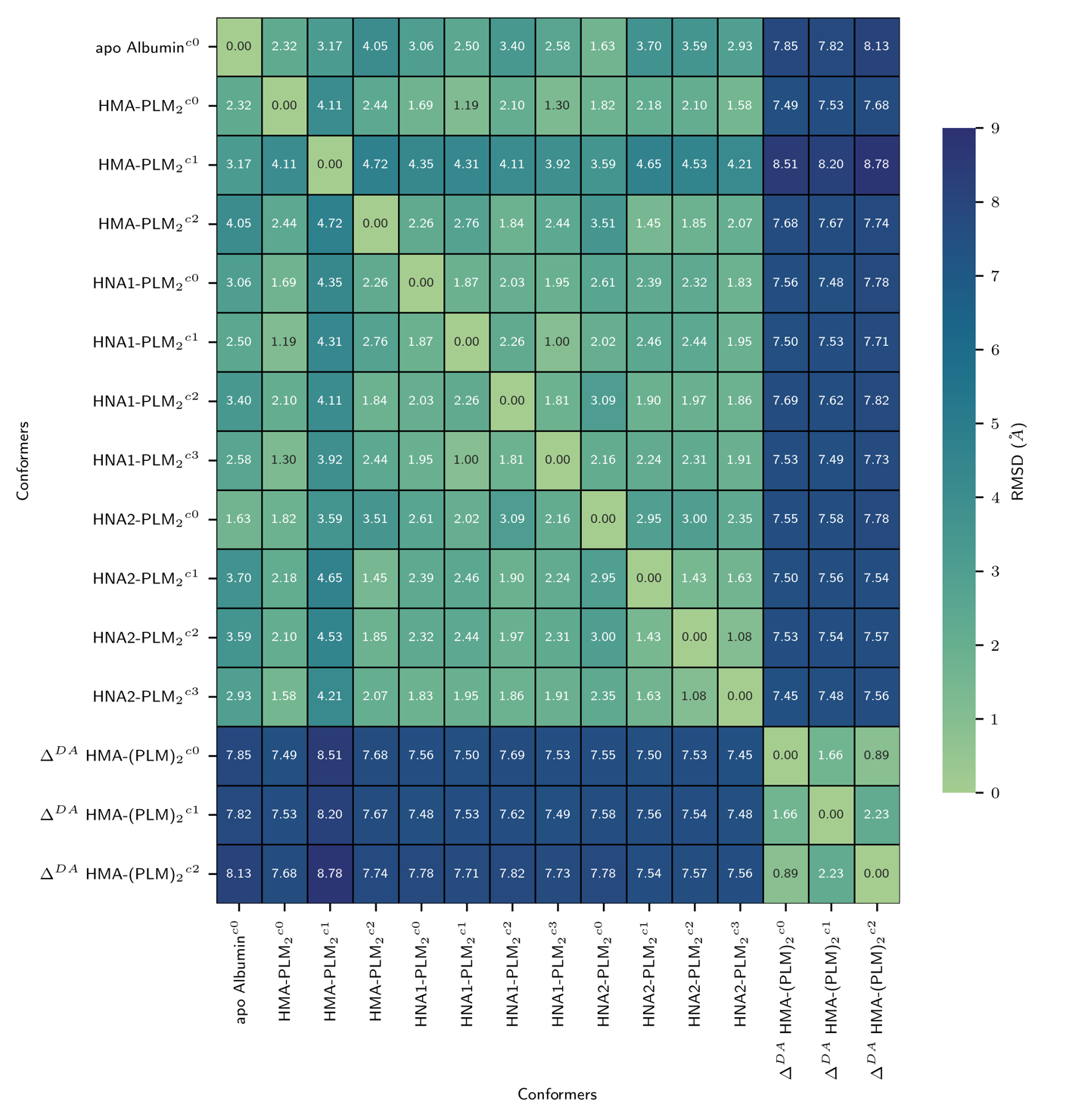


Supplementary Figure S6. Calculated backbone RMSD (Å) between all representative cluster snapshots identified from density peak clustering.


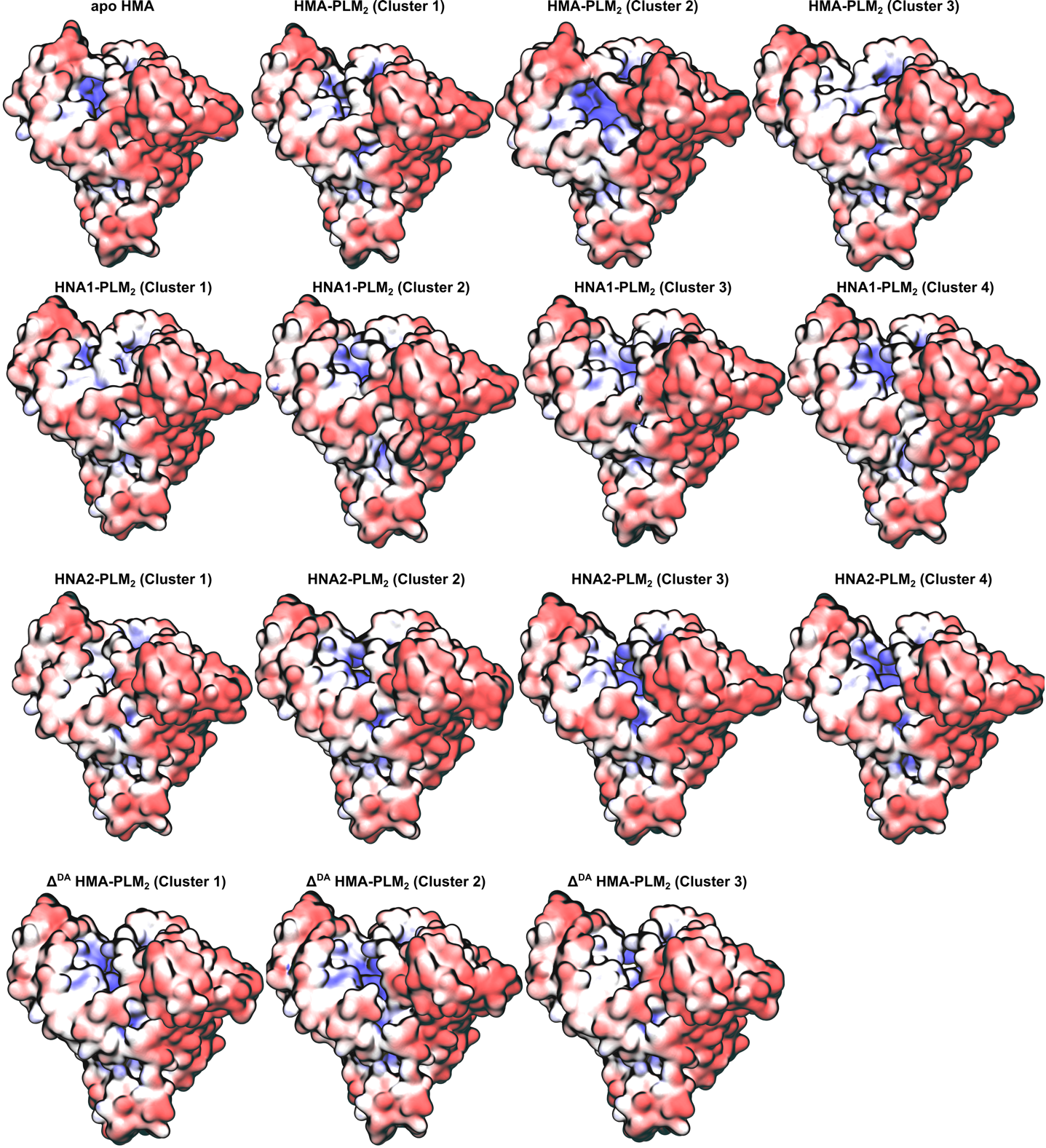


Supplementary Figure S7. Calculated electrostatic potentials for each cluster representative snapshots obtained during MD simulations.
